# Detection of Epstein-Barr virus in gastric adenocarcinoma: qPCR and FISH comparison

**DOI:** 10.1007/s00430-021-00724-3

**Published:** 2021-12-03

**Authors:** Igor Brasil-Costa, Carolina Rosal Teixeira de Souza, Iran Barros Costa, Liann Filiphe Pereira dos Santos, Luana César Ferraz Paixão, Alessandra Alves Polaro, Talita Antonia Furtado Monteiro, Rommel Mario Rodríguez Burbano

**Affiliations:** 1 Virology Section, Evandro Chagas Institute, Ananindeua, Pará 67030-000 Brazil; 2 Laboratory of Human Cytogenetics, Institute of Biological Sciences, Federal University of Pará, Belém, Pará 66075-110 Brazil; 3 Molecular Biology Laboratory, Ophir Loyola Hospital, Belém, Pará 66060-281 Brazil

**Keywords:** Gastric cancer, Epstein-Barr virus (EBV), qPCR, FISH, Methodological comparison, Brazil

## Abstract

EBV-associated gastric cancer accounts for about 10% of all gastric carcinomas worldwide. We aimed to verify the prevalence of EBV in gastric adenocarcinoma samples using FISH and qPCR and comparing the results obtained by both techniques. Gastric cancer samples from 191 cases were analyzed. The FISH assay was performed to detect small EBV RNAs (EBER1) and qPCR was performed to detect the EBV-EBNA-1 gene region. Cohen’s kappa index and the chi-square test were used to compare the methodologies and investigate correlations with the clinical-pathological data of the gastric adenocarcinoma patients. Most of the patients were men, and the average age was 60 years. The intestinal subtype cancer presented more aggressive stages with 90% of patients having a reactive FISH for EBV (EBV+), although the virus infection frequency in epithelial gastric tissue was only 1%. No positive association with clinicopathological features and EBV+ was found by FISH. Using qPCR analysis, the percentage of positive samples was lower (52.4%), and a positive association was found in samples from older patients (> 60 years). Interestingly, 71 qPCR-negative cases were detected by FISH in the presence of non-epithelial cells and in 10 qPCR-positive cases with no evidence of EBV according to FISH. The concordance between the two techniques was low, with only 57.6%. FISH is more informative for associating the gastric carcinoma with EBV positivity in tumor/epithelial cells; however, qPCR can provide relevant information regarding the progression and characteristics of neoplasia.

## Introduction

Approximately 20% of all cancer cases have a virus as an etiological factor [1]. In this context, Epstein-Barr virus (EBV) is associated with several types of malignant neoplasms, including gastric cancer. EBV-associated gastric cancer (EBVaGC) comprises about 10% of all gastric carcinomas worldwide [1, 2], affects more men, and globally ranks number one for deaths associated with the virus. The number of deaths linked to EBVaGC is exponentially proportional to age, primarily after 60 years [3].

EBV can reach the stomach through the saliva as a free virus particle in virus-infected B lymphocytes as well as infected oropharyngeal epithelial cells [4]. As the mucosal cells do not express CD21 (i.e., the classic EBV receptor typically found in B lymphocytes), these infected cells interact with other gastric cells [5]. EBV-positive tumors comprised 9% of all tumors classified by The Cancer Genome Atlas (TCGA) network [6].

The presence of EBV in a patient with gastric cancer was first described using polymerase chain reaction (PCR) in a case of undifferentiated lymphoepithelioma type gastric cancer, a histological type similar to nasopharyngeal lymphoepithelioma [7, 8]. The stomach is generally classified into two topographical subsites, the cardia (upper stomach) and noncardia (lower stomach) [9]. EBV loses infectivity more readily upon reaching the stomach, which may explain the higher prevalence of gastric cancer associated with the virus in the upper part of the organ [10]. Among all types of gastric cancer, adenocarcinomas account for about 95% of cases [9], and approximately 10% of these are associated with EBV [10]. The TNM Classification of Malignant Tumors (TNM) is internationally accepted for classifying this type of gastric cancer into stages I, II, III, and IV, reflecting an increasing order of severity, size, and malignancy [11, 12].

Imai et al. [13] analyzed 1,000 cases of gastric carcinomas randomly selected and, through the combined diagnosis of PCR and in situ hybridization (ISH), demonstrated the presence of the virus in 70 cases (7%). In a subsequent study, Sousa et al. [14] analyzed more than 30,000 samples of patients with stomach cancer and identified the presence of EBV DNA in 8% of these.

It is understood that in histological material, the methods of choice for demonstrating the presence of EBV are in situ hybridization (ISH) with labeled nucleic acid probes and the Southern blot test. However, quantitative polymerase chain reaction (qPCR) is still widely used, as it is a more sensitive detection methodology [15].

The aim of this study was to verify the prevalence of EBV in gastric adenocarcinoma samples by means of two methodologies widely used for virus detection in neoplastic samples, in addition to comparing the results with clinicopathologic variables.

## Methods

In the present study, two methods of direct detection of EBV nucleic acids were used: a) fluorescent in situ hybridization (FISH) for the detection of small EBV RNAs (EBER1) and b) quantitative polymerase chain reaction (qPCR) for detection of the EBV-EBNA-1 gene region.

### Study population, tumor samples, and clinical data

Gastric cancer samples were obtained between 1998 and 1999 from Ophir Loyola Hospital, which received patients from all over the state of Pará, Brazil. A total of 356 samples of gastric adenocarcinoma were obtained from paraffin-embedded tissue, and DNA was extracted from fresh tumors. Despite this, only 191 samples had both the DNA-PI-paraffined material; thus, they were used to compare the methodologies. Patients were not required to give informed consent to the study because the analysis used anonymous clinical data that were obtained after each patient agreed to treatment by written consent.

The samples were stored in the Laboratory of Human Cytogenetics of the Federal University of Pará (LCH/UFPA) until use. The epidemiological and clinicopathological characteristics of the patients were obtained through the analysis of medical records of the hospital and LCH/UFPA’s gastric tumor database. The study was forwarded and approved (opinion number 121.902 of September 27, 2012) by the Research Ethics Committee of the Evandro Chagas Institute in accordance with the norms regulating research involving human beings.

### EBV detection

For the FISH assay, small EBV RNAs (EBER1), which are highly expressed in cells with latent infection, were investigated in slides with approximately 5 μm of tissue. For this, the Clark Laboratories™ Kit was used according to the manufacturer’s instructions.

The DNA of formalin-fixed, paraffin-embedded (FFPE) tissues of gastric adenocarcinomas (4–5 slices of 5 μm of tissue) was obtained using a QIAamp DNA FFPE Tissue Kit (QIAGEN, Venlo, Netherlands), and DNA from fresh tissue (200 μg) was extracted using TRIzol^®^ (Thermo Fisher Scientific, Massachusetts, USA), following the manufacturer’s instructions. Then, the DNA was quantified with the Qubit^®^ dsDNA BR Assay kit on Qubit^®^ equipment (Thermo Fisher Scientific, Massachusetts, USA). The qPCR test was performed for the detection of the EBV-EBNA-1 gene region, using the qPCRAlert EBV^®^ Kit (NANOGEM), on the Rotor-gene Q^®^ equipment (QIAGEN, Venlo, Netherlands), with initial programming at 50 °C for 2 min for decontamination and 95 °C for 10 min for initial denaturation, followed by 45 cycles of 95 °C for 15 s for annealing and 60 °C for a 1 min extension. A standard curve (with controls with 10^5^, 10^4^, 10^3^, and 10^2^ copies) was used to verify the efficiency of the reaction, and ultrapure water was used as a negative control.

### Statistical analysis

The Shapiro–Wilk test was used to evaluate the distribution of the samples. The results of the analyses of infection by the two methodologies were correlated with the clinical-pathological data of the gastric adenocarcinoma patients using the Chi-square test. The comparison between methodologies for EBV detection was performed using Cohen’s kappa index. Considering ISH as the gold-standard method for EBV detection in tumor tissues, the sensitivity and specificity values were also calculated for PCR. A significance level of *P* < 0.05 was used for all analyses, and a 95% confidence interval was also applied. The results are given as the *P* value, odds ratio (OR), and confidence interval (CI). The statistical methods were performed on BioEstat v5.3 (https://www.mamiraua.org.br/downloads/programas/) and were reviewed by Carlos Eduardo de Melo Amaral, a biomedical statistician, from the Pará State Center for Hematology and Hemotherapy.

## Results

Of the 191 cases of gastric adenocarcinoma studied, the mean age of the patients at the time of sampling collection was 60 (min.: 28; max.: 89) and the majority were men (135; 70.7%). Nearly 55% (106/191) of the tumors were of the intestinal type according to Lauren’s criteria [16], and the tumors were distributed as follows in the stomach: 37,7% (72/191) cardia; 2.6% (5/191) fundus; 4.2% (8/191) body; 42.4% (81/191) antrum, and 13.1% (25/191) in more than one region, such as the body and antrum.

Regarding TNM classification and tumor staging, a heterogeneous distribution was observed (Table 1). The majority of the cases were aggressive: T3 or T4 (159/191; 83.2%) and had lymphonodal metastasis (N1, N2, or N3) (182/191; 95.3%), and almost half (45%) had distant metastasis. The percentage of cases per stage was 2.09% (4/191), 26.7% (51/191), 24.08% (46/191), and 45.03% (86/191) for stages I, II, III, and IV, respectively.

**Table 1:** TNM classification and staging of gastric adenocarcinoma cases studied

Staging	TNM	Number of cases	Percentage (%)
I	T1N1M0	2	1.05
	T2N0M0	2	1.05
	Subtotal	4	2.09
II	T2N1M0	2	1.05
	T2N2M0	4	2.09
	T3N0M0	3	1.57
	T3N1M0	42	21.99
	Subtotal	51	26.70
III	T2N3M0	3	1.57
	T3N2M0	25	13.09
	T3N3M0	1	0.52
	T4N1M0	5	2.62
	T4N2M0	10	5.24
	T4N3M0	2	1.05
	Subtotal	46	24.08
IV	T1N1M1	3	1.57
	T2N1M1	6	3.14
	T2N2M1	8	4.19
	T2N3M1	2	1.05
	T3N0M1	3	1.57
	T3N1M1	7	3.66
	T3N2M1	29	15.18
	T3N3M1	4	2.09
	T4N1M1	10	5.24
	T4N2M1	11	5.76
	T4N3M1	3	1.57
	Subtotal	86	45.03
Undefined	T3N0Mx	1	0.52
	T3N1Mx	1	0.52
	T3N2Mx	1	0.52
	T4N1Mx	1	0.52
	Subtotal	4	2.09
Total		191	100.00

Of the paraffin-embedded blocks involved in the project, 90% (171/190) had reactive FISH. However, only two samples showed fluorescent labeling in coat cells/epithelial cells, which characterizes the presence of EBV in the nucleus of adenocarcinoma tumor cells. The others presented labeling in non-epithelial cells, such as lymphocytes. Therefore, in the present study, the virus infection frequency in the epithelial gastric tissue from patients was found to be 1% (2/191) by FISH.

No positive association (*P* > 0.05) was found with EBV(+) by FISH technique and the variables gender, age, TNM classification, localization, and tumor histology. However, positive cases are more frequently seen in men aged ≥ 60 years old, in the proximal region of the stomach, in patients with lymph node metastasis, and in more advanced stages.

Using qPCR analysis, the percentage of positive samples found was lower than that found using FISH (52.4%; 110/191). The direct association of the qPCR results and the clinical-pathology variables studied by the chi-square test was only statistically significant for the age variable (*P* = 0.041, OR = 1.829, CI 1.023–3.267), and the virus was more frequently detected in carcinoma biopsies of older patients (Table 2) (mean of 62.25 vs. 57.45 for cases with undetectable virus result).

**Table 2:** Comparison of EBV positivity by qPCR with clinical-epidemiological variables

Clinical-epidemiological variables	EBV positive	EBV Undetectable	*P* value
Age			
≥ 60 years	64 (33.5)	35 (18.3)	**0.041**
< 60 years	46 (24.1)	46 (24.1)	
Gender			
Male	75 (39.3)	60 (31.4)	0.377
Female	35 (18.3)	21 (11)	
Location			
Proximal	45 (23.6)	27 (14.1)	0.286
Distal	65 (34)	54 (28.3)	
Histological type			
Intestinal	56 (29.3)	50 (26.2)	0.137
Diffuse	54 (28.3)	31 (16.2)	
Tumor aggressiveness			
T1 and T2	18 (9.4)	14 (7.3)	0.866
T3 and T4	92 (48.2)	67 (35.1)	
Lymph node metastasis			
N0	3 (1.6)	6 (3.1)	0.131
N1, N2 and N3	107 (56)	75 (39.3)	
Distant metastasis			
M0	57 (30.5)	44 (23.5)	0.692
M1	51 (27.3)	35 (18.7)	

Bold value indicates significant *P* value

The presence of metastases was not directly associated with infection. It was also observed that the number of positive qPCR cases was higher among patients with more severe staging (III and IV) (*P* > 0.05). The positivity for EBV in these more severe cancer cases was 73.4% (80/109).

**Fig. 1:**
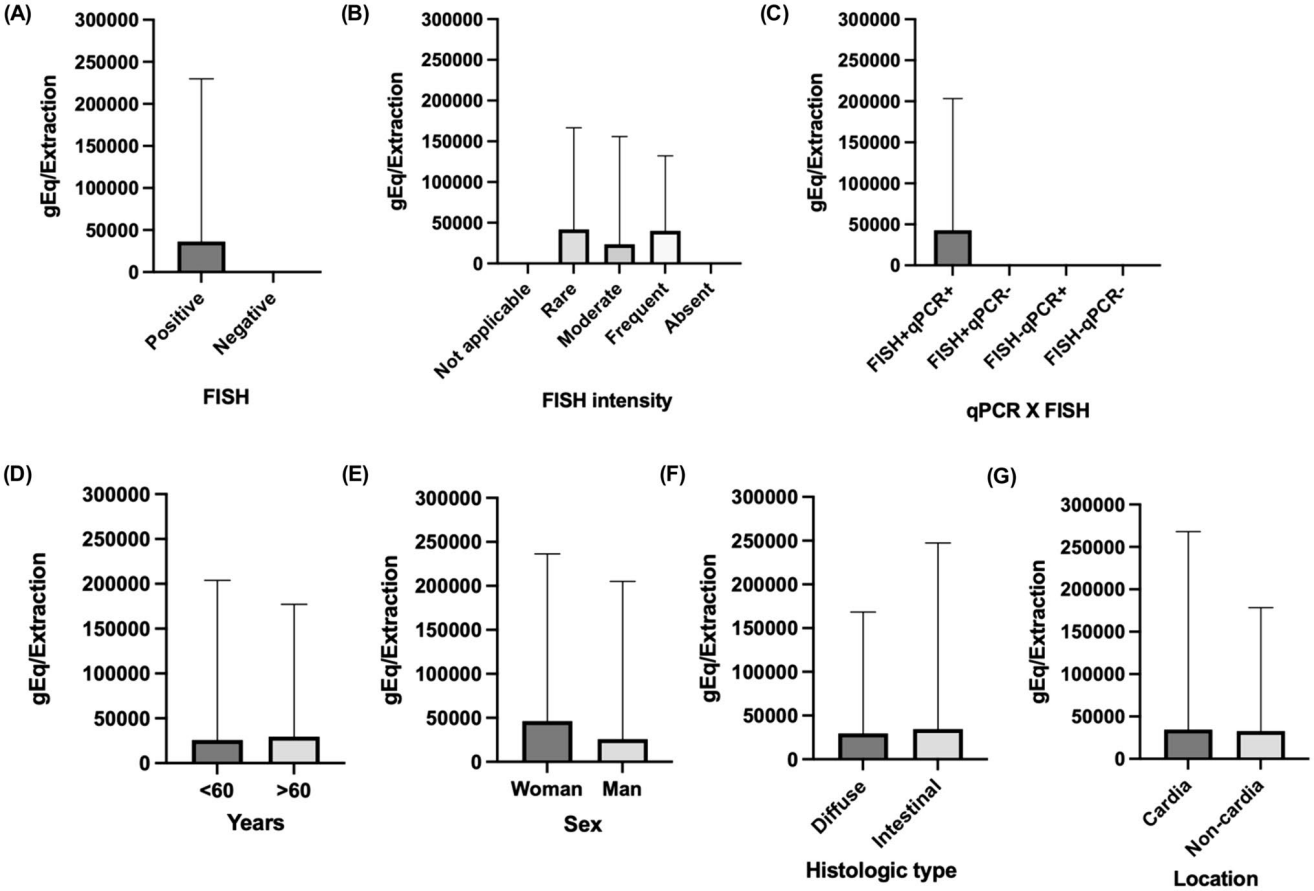
Distribution of viral load detected by qPCR and its relationship with FISH (**A**), FISH signal intensity (**B**) groups comparison (**C**), age (**D**), sex (**E**), tumor histologic type (**F**), and tumor location (**G**)

Interestingly, we found 71 PCR-negative cases with the presence of infected non-epithelial cells detected by ISH, and 10 PCR-positive cases with no evidence of EBV by FISH. The comparison of the two methodologies (FISH and qPCR) applied for the detection of EBV showed that they did not produce similar results (Table 3). Quantitative analyzes of viral genomic equivalent/extraction were performed and correlated with FISH results (including FISH labeling intensity) as well as age, sex, tumor histologic type, and location, but no significant associations were found (Fig. 1). The concordance between the two techniques was 57.6% (Cohen’s kappa index = 0.036; *P* > 0.05) [17]. In addition, the qPCR test had 58.5% sensitivity and specificity of 50%, which were considered low.

**Table 3:** Comparison between the results of the methodologies used to identify the EBV

	In situ hybridization (epithelial cells)	
qPCR	EBV (+)	EBV (−)
EBV (+)	2	108
EBV (−)	0	81
Total	2	189

## Discussion

It is well established that EBV has an important role as a conditioning agent for the appearance and evolution of gastric adenocarcinoma. The presence of the virus may deregulate the expression of several human genes related to the neoplastic process, especially those involved in the immune response [18, 19].

Regarding the pathological clinical features, both FISH and qPCR found more EBV in older men and in patients in a more advanced stage of cancer, reproducing the findings obtained by many authors around the world [4, 20, 21]. In addition, the distal region and intestinal type were the most affected. These results corroborate those of Souza et al. [21], who used a sample from the same state (Pará, Brazil) as the one used in this study. Nevertheless, the cases in which epithelial cells were shown to be infected by EBV using FISH belonged to the cardiac region, one was intestinal, and the other was a diffuse adenocarcinoma subtype. This finding agrees with other studies that point to the cardia and corpus as the gastric regions more associated with EBV(+) in ISH cases [5, 22, 23].

Some meta-analyses found a global prevalence of EBV ranging from 7.96 to 11.3% on average in patients with gastric cancer [10, 14, 24]. However, the frequency of EBV-positive gastric carcinoma varies widely worldwide, with the lowest prevalence in Europe and highest in America [14].

Considering only epithelial cell staining, the detection rate found by the FISH methodology in the present study was low (1.05%) compared to most EBV detection studies. Despite this, other studies showed frequencies in line with the results obtained—1.7% in the United Kingdom [25], 0% in England [26], and 1.3% in New Guinea [27].

In Brazil, studies using ISH observed frequencies that varied between 5% and 11.32%, with the highest percentage obtained in populations of the state of São Paulo [23, 28–31]. For the state of Pará, a percentage of EBV positivity for ISH of 9.6% was described [21].

In addition to the possible small technical variations, this large variation in the prevalence translates the differentiated condition of each population in relation to the susceptibility to EBVaGC and reinforces the multifactorial nature of this cancer. Although, apparently, this is not influenced by economic factors as there is a high variability within the same country and region.

Using the qPCR molecular tool, a detection rate of 52.4% (110/191) of EBV was found. Other studies have observed higher percentages, such as Ryan et al. [32], which detected 76.1% in North and Central America and Nogueira et al. [25], which observed 90.2% in Portugal using the qPCR methodology, and Aquino et al. [33], which found a prevalence of 80% in Manaus (city of the North of Brazil) using conventional PCR. However, contrasting results were verified by Martínez-López et al. [34] in Mexico and Lee et al. [35] in South Korea, with percentages of 10.67% and 10%, respectively, using conventional PCR. The large variation in the percentage values of virus detection by molecular biology can be attributed to the different sensitivities of the PCR or qPCR used in each study.

It is important to report that the PCR and qPCR methodologies identify the genetic material of the virus present in the DNA of the sample present in the tumor stroma, regardless of which cell the DNA belongs to (e.g., tumoral or B lymphocytes) [22]. Additionally, the qPCR(+) results with FISH(−) staining in any cell can occur because the qPCR detects a small number of infiltrating EBV-positive lymphocytes that may not be detected by FISH since the amount of tissue used to extract DNA for qPCR is greater than the sections used for FISH. To minimize this difference, we also tried to correlate the viral genomic equivalent quantity/extraction (Fig. 1); however, no association was found. Other works have been successful in demonstrating the association between EBV viral load and disease severity [36, 37].

The greater the number of circulating EBV-infected B lymphocytes, the easier it is to find access to gastric tissue and, consequently, the easier it is for it to be detected by molecular biology methods, such as qPCR, in tumor tissue [38]. At the cellular level, patients with gastric cancer have decreased numbers of CD3+ and CD8+ cells and an increase in CD4+, CD19+, CD44+, CD25+, and NK compared to a disease-free control [39]. The greater proliferation of CD19+ cells increases the chances of reactivation of EBV since it is the cell for which the virus has a higher affinity [40]. According to the clonal proliferation of these cells, viral load may be more representative.

In the present study, the comparison of the results obtained from FISH and qPCR showed fair concordance (Cohen’s kappa index = 0.036) [17]. In addition, the qPCR test had 58.5% sensitivity and specificity of 50%, which are considered low. The difference between the results of the two methodologies is mainly due to the existence of other infected cells such as lymphocytes in the stroma. The fact that some cases were positive using one technique but negative using another—especially those shown as negative cases by FISH but positive by qPCR—may be due to the use of a small and thin slice of tumor tissue for FISH analysis and a greater amount of tumor material for the extraction of genetic material for qPCR. This could have increased the quantity of cells analyzed and potentially infected. Still, Ryan et al. [32] identified a few polymorphisms in the EBV genome, which leads to difficulties in PCR amplification of some viral genes in a few cases, which could explain the qPCR(−) cases with FISH(+). However, the outcome of FISH is more informative in associating gastric carcinoma with the presence of EBV in tumor/epithelial cells [41, 42]; nevertheless, qPCR can provide relevant information regarding the progression and characteristics of the neoplasia.

The definition of the best technique for identifying EBV in gastric cancer is important since EBVaGC has a distinct tumorigenic profile. In addition, it presents the opportunity for using EBV as a potential biomarker for treatment.

## Conclusion

FISH was more informative for associating gastric carcinoma with EBV positivity in tumor/epithelial cells. Additionally, the results suggest that a greater number of cuts or more automated methodologies such as flow cytometry may be used. New research should be encouraged to uncover the role of infected B lymphocytes in gastric carcinogenesis.

*Core tip* The presence of Epstein-Barr virus (EBV) was investigated by two different methodologies, qPCR and FISH, in gastric adenocarcinomas, and their relationship with the clinicopathological characteristics of these patients. The agreement between the two methodologies was low. FISH was more informative for associating gastric carcinoma with the presence of EBV in tumor/epithelial cells, although qPCR can demonstrate the presence of EBV even before it enters a latent state. The only positive association found was with older patients and qPCR + cases.
